# Electroencephalography Reflects User Satisfaction in Controlling Robot Hand through Electromyographic Signals

**DOI:** 10.3390/s23010277

**Published:** 2022-12-27

**Authors:** Hyeonseok Kim, Makoto Miyakoshi, Yeongdae Kim, Sorawit Stapornchaisit, Natsue Yoshimura, Yasuharu Koike

**Affiliations:** 1 Swartz Center for Computational Neuroscience, Institute for Neural Computation, University of California San Diego, La Jolla, CA 92093, USA; 2 Department of Industrial Engineering and Economics, Tokyo Institute of Technology, Tokyo 152-8550, Japan; 3 Department of Information and Communications Engineering, Tokyo Institute of Technology, Yokohama 226-0026, Japan; 4 Institute of Innovative Research, Tokyo Institute of Technology, Yokohama 226-0026, Japan

**Keywords:** electromyography, electroencephalography, satisfaction, subjective response, robot control

## Abstract

This study addresses time intervals during robot control that dominate user satisfaction and factors of robot movement that induce satisfaction. We designed a robot control system using electromyography signals. In each trial, participants were exposed to different experiences as the cutoff frequencies of a low-pass filter were changed. The participants attempted to grab a bottle by controlling a robot. They were asked to evaluate four indicators (stability, imitation, response time, and movement speed) and indicate their satisfaction at the end of each trial by completing a questionnaire. The electroencephalography signals of the participants were recorded while they controlled the robot and responded to the questionnaire. Two independent component clusters in the precuneus and postcentral gyrus were the most sensitive to subjective evaluations. For the moment that dominated satisfaction, we observed that brain activity exhibited significant differences in satisfaction not immediately after feeding an input but during the later stage. The other indicators exhibited independently significant patterns in event-related spectral perturbations. Comparing these indicators in a low-frequency band related to the satisfaction with imitation and movement speed, which had significant differences, revealed that imitation covered significant intervals in satisfaction. This implies that imitation was the most important contributing factor among the four indicators. Our results reveal that regardless of subjective satisfaction, objective performance evaluation might more fully reflect user satisfaction.

## 1. Introduction

In the development of an advanced human–robot interface, user satisfaction should be investigated to determine the optimal control configuration. Several studies have evaluated interface usability [1,2,3] and user satisfaction [4]. For an individualized interface, the system must recognize the reactions of the user to its responses. Although some studies have investigated brain-related user responses, such as brain activity reflecting delayed response during real-time cursor control [5], brain activity during control for interfacing has rarely been investigated regarding subjective feelings. Brain activity related to a variety of subjective feelings has been investigated based on self-reported information. For example, brain activity with respect to changes due to the mood of a film was investigated in [6]. Changes in emotional states induced by auditory stimuli were investigated using electroencephalogram (EEG) signals [7], and emotional states related to music were investigated using EEG data [8]. The use of EEG signals to detect emotion has been validated by several classification methods such as support vector machine, k-nearest neighbor, naive Bayes, long- and short-term memory, and deep belief networks (DBNs) [9]. In addition, because EEG signals have been involved in a variety of analysis methods, such as fuzzy decision tree [10], and combined with other sensors [11], they can be adopted for subjective evaluation of human–robot interfaces.

Users control a robot for a purpose (e.g., to grasp an object). If they fail to control the robot or feel that it might not grasp the object, they feed a different input into the robot. In such cases, when a user gives up or succeeds in moving the robot, defining the user’s satisfaction in a trial is necessary. To track the source of the satisfaction, first, we need to understand the moment that dominates the satisfaction and the factors of robot movement that induce user satisfaction. The representation of objective indicators of robot movement by the brain has not been elucidated. This could be linked to the user’s evaluation of the robotic performance, which is information that could be exploited.

However, the timing of and the factors that affect satisfaction remain unclear. Satisfaction can be measured by subjective responses to a questionnaire. We attempted to determine brain activity related to satisfaction and the important interval, which dominates satisfaction, according to the difference between EEG in unsatisfactory and satisfactory tasks. For factors that cause satisfaction, we selected four indicators (stability, imitation, response time, and movement speed) related to the robot’s performance to determine the extent to which these performance-related indicators, which might be independent of satisfaction, are relevant to satisfaction. We categorized performance indicators as abstract and direct indicators. By determining parameters that the robot system can handle, we can focus on the parameters of satisfaction. Otherwise, we should determine abstract concepts for performance evaluation that primarily contribute to satisfaction within the brain. However, if we cannot use abstract parameters for robot control, they can be decomposed for easy handling. Unlike speed and time, stability and imitation were intended to represent abstract indicators. This enables us to determine whether satisfaction can be attributed to directly controllable indicators. The subjective responses obtained from the questionnaire can be linked to EEG signals to determine relevant brain areas and are observed in the same region as satisfaction.

This study investigated aspects of both satisfaction and EMG-based robotic control. We designed a system controlled by EMG signals. In each trial, we exposed the participants to different experiences by changing the cutoff frequencies (1, 2, 5, and 10 Hz) of a low-pass filter for the input EMG signals. The participants attempted to grab a bottle by controlling the robot. At the end of each trial, the participants were asked to complete a questionnaire to evaluate the four indicators and their satisfaction levels. The EEG signals of the participants were recorded while they controlled the robot and responded to the questionnaire. We further compared the brain activities based on five parameters (the aforementioned four indicators and the level of satisfaction).

## 2. Experimental Procedures

### 2.1. Participants

A group of eight healthy individuals comprising five men and three women participated in the experiment. The mean and standard deviation of their ages were 26.75 years and 3.33 years, respectively. All participants were right-handed and provided written informed consent before the experiment. This study was conducted as per the Declaration of Helsinki and approved by the ethics committee of the Tokyo Institute of Technology (ethics number: 2019002).

### 2.2. Experimental Apparatus and Data Collection

**Figure 1:**
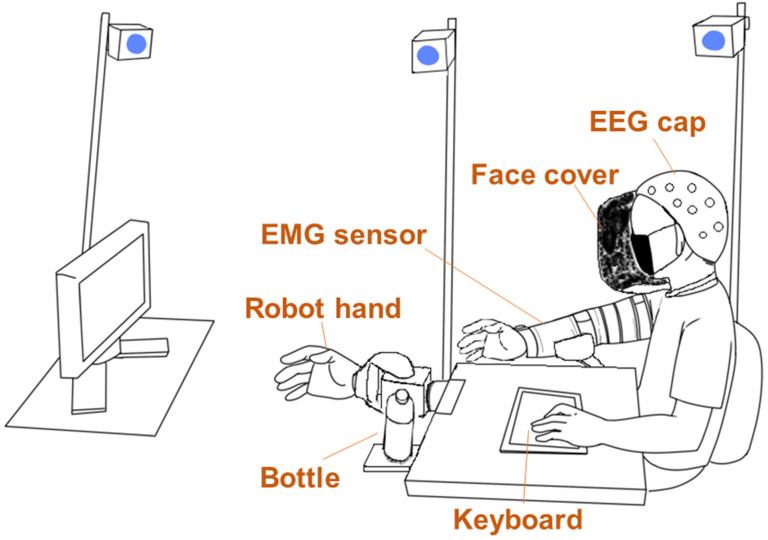
Experimental environment (not to scale). During the experiment, participants sat on a chair and placed their right arm on an armrest attached to the table. A keyboard was placed on the table through which participants responded to questions by pressing a button. For the task, participants were asked to grab a bottle, which was positioned such that the robot hand could be bent to grab the bottle. The robot hand could bend/extend a wrist and grip fingers with one degree of freedom (DOF). An opaque face cover prevented the participants from seeing their right arm. A monitor was placed in front of the table along the midline of the body, which includes a robot hand on the line to enable participants to easily see the screen.

Figure 1 shows the experimental environment. A controllable robotic hand (qb SoftHand, qbrobotics, Navacchio, Italy) was fixed in front of a table. The robot provides 19 anthropomorphic DOFs, one synergy, one motor, 1.7 kg of nominal payload, 0.77 kg of weight, and 1.1 s for clenched fists from a wide-open position. The bottle was placed near the robotic hand. During the experiment, the participants sat on a chair and wore an EEG cap. The sitting posture enabled them to see the robotic hand and screen. Their left hand was placed on a keyboard to answer the questionnaire, and their right arm was placed on an armrest. To fix an EMG sensor, an arm brace was worn on their right hand. In addition, the participants wore a face cover with opaque paper, which prevented them from seeing their right hand. EMG signals from 32 channels were measured with an array EMG sensor [12] and 24 bit resolution at a sampling rate of 500 Hz and used for robotic control. An OptiTrack motion capture system (NaturalPoint, Inc., Corvallis, OR, USA) recorded the motions of the participants and robotic hand. Three motion capture markers were attached to the back of the wrist on the end of the radius/ulna, back of the hand on the third metacarpal bone, and back of the middle finger (between the metacarpophalangeal and proximal interphalangeal joints) of the participants and the robot. EEG signals were recorded from 64 electrodes using a BioSemi ActiveTwo system (BioSemi, Amsterdam, Netherlands) with 24 bit resolution at a sampling rate of 512 Hz, as shown in Table 1.

### 2.3. Experimental Paradigm

Calibration was necessary because the robotic hand was controlled using only EMG signals. First, each participant sat on a chair and placed their right arm on the armrest, as shown in Figure 1. Then, they were instructed to perform six different hand motions: hand gripping/opening, wrist flexion/extension, and pronation/supination. Each hand motion was repeated thrice. The EMG signals measured during the motions were rectified and filtered with a second-order Butterworth low-pass filter with a cutoff frequency of 5 Hz. Noisy channels were rejected before preprocessing. By using the hierarchical alternating least squares algorithm [13], we extracted two muscle synergies from the EMG signals involved in the gripping/opening motions and four muscle synergies from those involved in the other motions. If the extracted muscle synergies did not reflect the expected movement, the number of synergies was increased. The weight of each muscle synergy was normalized to the maximum weight. Next, the joint angles were estimated using a musculoskeletal model [14] and introduced into the robot hand as an input command. After calibration, the participants learned to control the robot hand. They were subsequently instructed to remember and replicate their methods of controlling the robot hand during the task. However, if a participant failed to control the robot, the calibration process was repeated.

In the experiment, we used four cutoff frequencies (1, 2, 5, and 10 Hz) of the low-pass filter of the EMG signals. The participants were exposed to different control experiences. Each trial had one of the four cutoff frequencies, which were determined randomly. The participants were not informed of the aspects that were changed or the various options available. They were only informed that they would have different control experiences in each trial. At the beginning of the experiment, a “wait” message appeared on the screen, and the participants immediately put their hands in a resting state. When the message was changed to “Go”, the participants attempted to grab the bottle by controlling the robot. This was achieved by flexing their wrists and grasping their hands. When the robot hand approached the bottle tracked by motion capture markers, a questionnaire appeared on the screen after 2 s. However, this information was concealed from the participants. Notably, no trial in which the interval between the “Go” cue and the questionnaire was less than 2 s occurred. The participants were asked to evaluate the following four indicators related to the control performance: stability (unstable vs. stable), imitation (bad vs. good), response time (delay vs. no delay), and movement speed (extremely slow vs. extremely fast). Stability and imitation were selected as more abstract indicators than speed and time to determine whether satisfaction was attributed to directly controllable indicators. After answering the survey, they were asked to evaluate their subjective satisfaction, regardless of the objective performance of the robot. The questionnaire was designed using a five-point Likert scale. When they completed the questionnaires, a “relax” message appeared on the screen until the next trial began. This procedure was subsequently repeated. The indicators are defined as follows: stability refers to the extent to which the robot shakes unnecessarily or performs unnecessary movement during the trial, imitation is determined by the extent to which the robot’s movement is identical to the movement of the actual hand, response time refers to the time required to move the robot, movement speed is determined by the speed of the robot during the trial, and satisfaction is determined by the extent of the participants’ feelings about the trial if they used this interface as a user.

The participants were required to perform three runs, with each run consisting of 81 trials and a break between successive runs. For each run, a frequency of 10 Hz was presented 21 times, and the other frequencies were presented 20 times each. Participant 6 performed two runs, and participant 1 performed one run consisting of 97 trials (25 trials for the 10 Hz and 24 trials for each of the others).

### 2.4. EEG Analysis

EEGLAB was used for preprocessing [15] as follows: EEG data were resampled at a frequency of 256 Hz and filtered using a high-pass filter with a cutoff frequency of 1 Hz. We used cleanLineNoise [16] to remove line noise and artifact subspace reconstruction (ASR) to correct the artifacts [17,18]. The cutoff parameter for the ASR was set to 10. Cleaned data were re-referenced to the average. Next, we performed an independent component analysis using Adaptive Mixture ICA. An equivalent dipole model corresponding to each independent component was fitted using fitTwoDipole [19]. All independent components were identified using ICLabel [20].

The components identified as the brain were used for group-level analysis, for which k-means clustering was performed based on the dipole locations of the brain components. Ten clusters were determined using the silhouette index [21]. We extracted epochs between 0 s (“Go” cue) and 2 s after the “Go” cue to calculate the event-related spectral perturbations (ERSPs) of the independent components within a frequency of 50 Hz with intervals of 1 Hz using the Morse wavelet. For comparison, the various responses to the questionnaire were classified into two groups such that their proportions were approximately 50% each. For statistical testing, we performed a cluster-based permutation test with a weak control of the family-wise error rate [22]. Here, the number of permutations was set to 5000, and the threshold *p*-value for preselection was set to 0.01. Generally, the minimum and maximum values for a permutation within each cluster are selected for multiple testing corrections for comparison. However, we selected the minimum (or maximum) of the minimum (or maximum) values obtained from all the clusters for each permutation to determine the 5th and 95th percentile values, respectively. These values were commonly applied to achieve a multiple testing correction, which was a stronger correction than usual for the clusters.

## 3. Results

**Figure 2:**
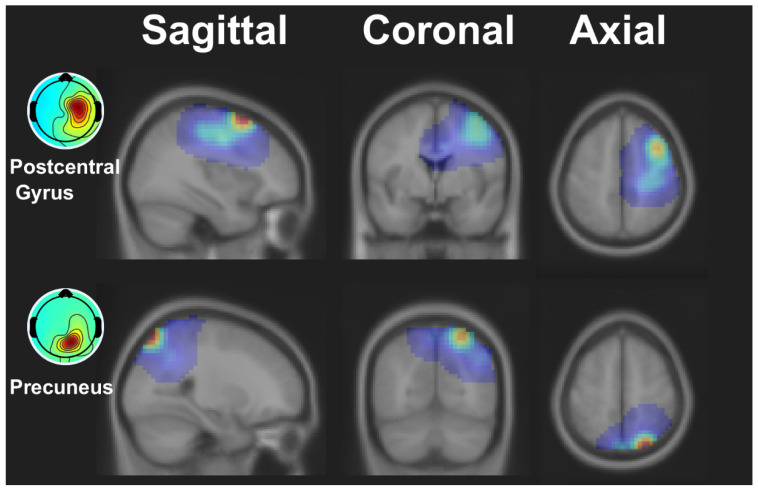
Dipole densities of clusters showing significant differences between conditions. The mean Montreal Neurological Institute (MNI) coordinate of the first cluster was (35 0 52), and the estimated location was the postcentral gyrus, with a probability of 17.9%. The mean MNI coordinate of the second cluster was (26 −69 55), and the estimated location was the precuneus, with a probability of 22.5%.

We obtained two clusters that were primarily related to the four indicators and satisfaction. Figure 2 illustrates the dipole densities of the clusters that exhibit significant differences. The anatomical regions estimated according to the dipole locations were the precuneus, with a probability of 22.5%, and the postcentral gyrus, with a probability of 17.9%.

**Figure 3:**
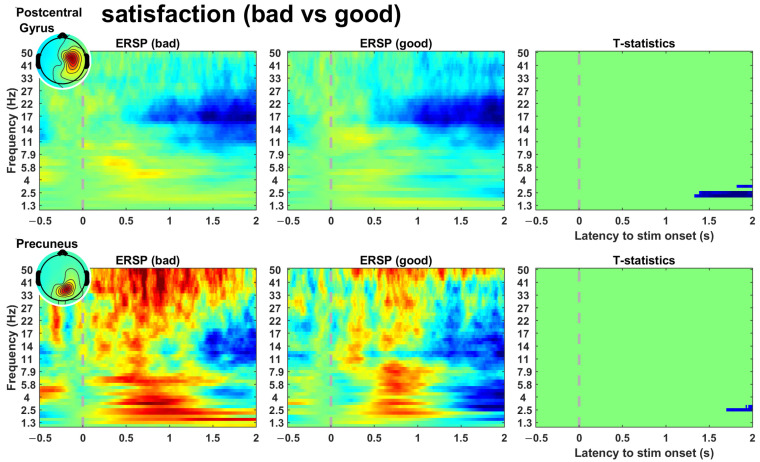
Event-related spectral perturbation of clusters including a significant area in the comparison of satisfaction. The dotted line represents the onset of the “Go” cue. In each figure set, the third column represents t-statistics and the significant area. This plot indicates that satisfaction is determined dominantly in the final phase of control.

**Figure 4:**
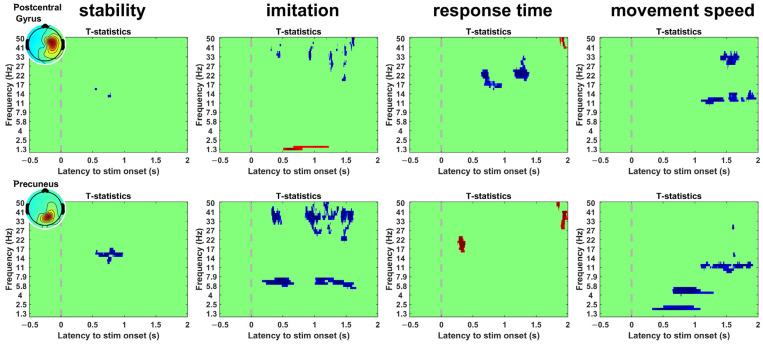
Significant areas in the comparisons; stability (unstable vs. stable), imitation (bad vs. good), response time (delayed vs. no delay), and movement speed (extremely slow vs. extremely fast). The dotted line represents the onset of the “Go” cue. The blue areas represent negative t-statistics, and the red areas represent positive t-statistics.

Figure 3 shows the ERSPs and t-statistics of the clusters in the comparison of satisfaction. The clusters exhibited significant areas that started from approximately 1.5 s and were sustained to the end of the epoch. Additionally, significant areas in the delta band power (1–4 Hz) were commonly found in both clusters. Figure 4 shows the significant areas of the clusters in the comparisons. An independent pattern of significant areas was identified in all comparisons. The cluster in stability exhibited a significant area within the range of approximately 0.5–1 s, and the clusters in the other indicators exhibited significant areas over the entire epoch. In addition, we observed common significant areas in both clusters. In the imitation comparison, significant areas in the gamma band power (30–50 Hz) were commonly found in both clusters. In the speed-of-movement comparison, significant areas in the alpha band power (8–13 Hz) were commonly found in both clusters. Table 2 shows the t-statistics of the significant areas. If both increasing and decreasing significant areas were found in a cluster, the values were calculated separately.

**Figure 5:**
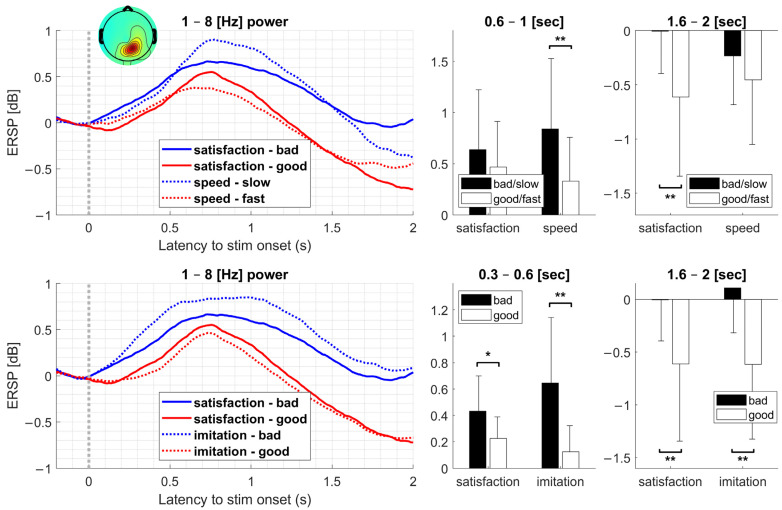
Power (1–8 Hz) of the clusters related to the precuneus. We extracted powers within the range of 1–8 Hz shown as a significant area in ERSP. The comparison of the movement speed exhibited a power difference within the range of 0.6–1 s, although the power difference in satisfaction was not significant. Although the power difference within the range of 1.6–2 s was significant in the comparison of satisfaction and imitation, the difference in the movement speed was not. Within the range of 0.3–0.6 s, satisfaction exhibited a moderate difference, and imitation exhibited a more significant difference. All tests were performed by *t*-test (*: *p* < 0.05; **: *p* < 0.01).

As satisfaction exhibited a significant difference in the low-frequency band, we checked event-related potential in the comparisons with significant differences over the frequency range, i.e., movement speed and imitation. Figure 5 shows the power of the clusters related to the precuneus in the range of 1–8 Hz. Within the range of 0.6–1 s, the comparison of the movement speed exhibited a power difference (*t*-test; *p* < 0.01), whereas that in the satisfaction was not significant. Within the range of 0.3–0.6 s, satisfaction exhibited a moderate difference (*t*-test; *p* < 0.05), and imitation exhibited a more significant difference (*t*-test; *p* < 0.01). Within the range of 1.6–2 s, the comparisons of both satisfaction and imitation exhibited significant differences (*t*-test; *p* < 0.01), whereas the difference in movement speed was not significant.

## 4. Discussion

By using the participants’ responses to the questionnaire, we investigated the reflection of brain activity on user satisfaction alongside performance indicators of EMG-based robot control. We found that two clusters primarily linked satisfaction and the indicators, as shown in Figure 2. Brain activity exhibited significant differences in satisfaction at a later stage but not immediately after feeding an input, as shown in Figure 3. The indicators exhibited their independent significant patterns in ERSPs, as shown in Figure 4.

User satisfaction exhibited significant differences primarily at the end of the epoch. This could imply that the level of satisfaction was primarily determined by the latest information, regardless of the robotic performance immediately after an input command. This might be a specific characteristic of satisfaction in the brain determined by the latest information. This has rarely been discovered in a study on user satisfaction in human–robot interaction [23]. However, a previous study on user satisfaction with a haptic interface reported that EEG power in the early period exhibited a significant correlation with user satisfaction [24]. The reported results revealed that each participant exhibited different frequency bands (alpha, delta, and high gamma), primarily contributing to satisfaction. We conjecture that the experiment in the previous study asked participants only about satisfaction, which would have different meanings depending on the individual. In our questionnaire, we suggested multiple indicators, which might have enabled the participants to limit their interpretation of satisfaction. In addition, different interface environments could define different satisfaction levels. In a dial interface, gamma EEG over the frontal area in the early period exhibited a significant contribution to satisfaction [25]. Additionally, some studies have reported that different paradigms exhibited different preferences in a brain–computer interface using motor imagery [26].

Among the four indicators, stability exhibited a significant difference in a portion of the epoch. However, the other indicators exhibited significant differences in most of the epochs. Several independent activations appeared to be involved in significant areas in response time and movement speed. The cluster related to the precuneus exhibited an earlier and more significant difference than the other clusters, indicating that movement speed and delay might be processed as information about a fundamental concept through the precuneus within the brain. As visual information is processed in two pathways, this might reflect the process on the dorsal pathway. Humans can intuitively recognize delay and speed immediately as they observe a robotic movement. However, considerable time is required to recognize stability, indicating that stability is represented as integrated information in the brain. Another high-level concept is the sense of agency, which refers to the feeling of control [27] and is dependent on delay and speed. Delay has been used to reduce the sense of agency [28]. A previous study reported that the speed of controlled objects is also related to the sense of agency [29]. This supports the idea that delay and speed are the fundamental factors affecting subjective feelings. Although delay appears to be a low-level concept, its effect is complex. Participants sometimes fail to perceive the correct delay [5]. Moreover, the awareness between certainty and uncertainty with respect to the delayed response of a robotic hand can induce a significant difference in the theta band of the parietal lobe [30]. Additionally, although unlike delay, imitation is not a simple concept, significant differences were observed immediately after the onset. This might be caused by differences in the gamma band related to sensory awareness [31,32], which may have influenced subjective feelings. The significant gamma powers in the other interval might be related to emotional processing [33].

In all the indicators, including satisfaction, the pattern of significant regions in one of the clusters resembled the pattern of the other cluster in the time–frequency plot, implying that the two clusters may be functionally connected and reflect information flow between them. Moreover, the gamma power in imitation, gamma power at the end of the epoch in the response time, and alpha power in the movement speed exhibited a larger difference area in the precuneus-related clusters than in the postcentral-gyrus-related cluster. Furthermore, user satisfaction exhibited a longer and larger significant area in the postcentral gyrus than in the precuneus. Considering the visual information pathway through the parietal area, satisfaction is primarily determined by other integrated information, unlike information through the precuneus at that time.

We used a k-means algorithm for clustering based on the dipole locations. However, several other methods could be used for clustering, such as hierarchical clustering or a sequential algorithm of partitional clustering. In this case, other features could be included for optimized clustering besides dipole location. These features should be investigated in future studies so that optimized components related to the task or stimulus can be extracted.

As the ERSPs in the low-frequency band were related to satisfaction, we compared them with imitation and movement speed, which exhibited significant differences. The speed indicator showed that the difference between powers within the range of 0.6–1 s was significant, whereas satisfaction exhibited a significant difference within the range of 1.6–2 s. This may imply that speed is not a primary contributor to satisfaction. For imitation, powers within the ranges of 0.3–0.6 s and 1.6–2 s exhibited significant differences. Satisfaction also exhibited significant differences in both intervals, but the difference in the early period was moderate. As imitation covered significant intervals in satisfaction, it was the most important contributing factor among the four indicators. We presented the indicators as evaluation indicators regardless of the participant’s satisfaction. Our results reveal that objective performance evaluation, regardless of subjective satisfaction, can fully reflect satisfaction. As no optimal control configurations exist, the robot system should evaluate the user’s satisfaction without measuring satisfaction. In other words, satisfaction should be estimated using information that the robot system can exploit. Our results prove the feasibility of this method with brain-related signals. However, the information that contributed to satisfaction was not a simple parameter such as delay or movement speed. The fundamental unit satisfaction processing in the brain might be neither of these factors. Although we have yet to elucidate the representation of imitation in the brain, we discovered integrated information related to a robot’s objective movement, causing satisfaction to be processed in the brain. In future studies, integrated information, which might consist of basic parameters such as delay and movement speed, should be investigated to ensure the decomposition of integrated information and determine more direct contributors to satisfaction. Then, the information could be exploited to determine individualized optimal parameters and used to generalize individualized preferred robot configurations.

## Figures and Tables

**Table 1:** Electrodes and description of electrode labels.

Label	Meaning
Fp	Prefrontal lobe
F	Frontal lobe
T	Temporal lobe
P	Parietal lobe
O	Occipital lobe
C	Central lobe
Combination of above labels	Position between two places
Even numbers	The right hemisphere
Odd numbers	The left hemisphere
Z	The midline on the coronal plane
Used electrodes	Fp1, Fp2, Fpz, AF3, AF4, AF7, AF8, AFz, F1, F2, F3, F4, F5, F6, F7, F8, Fz, FT7, FT8, FC1, FC2, FC3, FC4, FC5, FC6, FCz, C1, C2, C3, C4, C5, C6, Cz, T7, T8, TP7, TP8, CP1, CP2, CP3, CP4, CP5, CP6, CPz, P1, P2, P3, P4, P5, P6, P7, P8, P9, P10, Pz, PO3, PO4, PO7, PO8, POz, O1, O2, Oz, and Iz

**Table 2:** T-statistics of significant areas.

Indicator	Cluster	Mean	Min (or Max)
Stability	Precuneus	−3.79	−5.83
Imitation	Postcentral (increase)	3.26	3.89
	Postcentral (decrease)	−3.49	−6.15
	Precuneus	−3.01	−7.85
Response time	Postcentral (increase)	3.45	4.28
	Postcentral (decrease)	−3.8	−6.81
	Precuneus	3.99	6.66
Speed	Postcentral	−3.63	−5.73
	Precuneus	−3.79	−5.42
Satisfaction	Postcentral	−3.69	−4.30
	Precuneus	−3.22	−3.49

## Data Availability

The data presented in this study are available on request from the corresponding author.

## References

[1] Connan M., Ruiz Ramírez E., Vodermayer B., Castellini C. (2016). Assessment of a wearable force- and electromyography device and comparison of the related signals for myocontrol. Front. Neurorobotics.

[2] Barszap A.G., Skavhaug I.-M., Joshi S.S. (2016). Effects of muscle fatigue on the usability of a myoelectric human-computer interface. Hum. Mov. Sci..

[3] Belyea A., Englehart K., Scheme E. (2019). FMG Versus EMG: A Comparison of Usability for Real-Time Pattern Recognition Based Control. IEEE Trans. Biomed. Eng..

[4] Poritz J.M.P., Taylor H.B., Francisco G., Chang S.-H. (2020). User satisfaction with lower limb wearable robotic exoskeletons. Disabil. Rehabil. Assist. Technol..

[5] Kim H., Yoshimura N., Koike Y. (2020). Investigation of Delayed Response during Real-Time Cursor Control Using Electroencephalography. J. Healthc. Eng..

[6] Dennis T.A., Solomon B. (2010). Frontal EEG and emotion regulation: Electrocortical activity in response to emotional film clips is associated with reduced mood induction and attention interference effects. Biol. Psychol..

[7] Lee M., Shin G.-H., Lee S.-W. (2020). Frontal EEG asymmetry of emotion for the same auditory stimulus. IEEE Access.

[8] Plourde-Kelly A.D., Saroka K.S., Dotta B.T. (2021). The impact of emotionally valenced music on emotional state and EEG profile: Convergence of self-report and quantitative data. Neurosci. Lett..

[9] Wang J., Wang M. (2021). Review of the emotional feature extraction and classification using EEG signals. Cogn. Robot..

[10] Rabcan J., Levashenko V., Zaitseva E., Kvassay M. (2020). Review of methods for EEG signal classification and development of new fuzzy classification-based approach. IEEE Access.

[11] Callejas-Cuervo M., González-Cely A.X., Bastos-Filho T. (2020). Control systems and electronic instrumentation applied to autonomy in wheelchair mobility: The state of the art. Sensors.

[12] Koike Y., Kim Y., Stapornchaisit S., Qin Z., Kawase T., Yoshimura N. (2020). Development of Multi-sensor Array Electrodes for Measurement of Deeper Muscle Activation. Sens. Mater..

[13] Cichocki A., Phan A.-H. (2009). Fast local algorithms for large scale nonnegative matrix and tensor factorizations. IEICE Trans. Fundam. Electronics. Commun. Comput. Sci..

[14] Kawase T., Sakurada T., Koike Y., Kansaku K. (2017). A hybrid BMI-based exoskeleton for paresis: EMG control for assisting arm movements. J. Neural Eng..

[15] Delorme A., Makeig S. (2004). EEGLAB: An open source toolbox for analysis of single-trial EEG dynamics including independent component analysis. J. Neurosci. Methods.

[16] Bigdely-Shamlo N., Mullen T., Kothe C., Su K.-M., Robbins K.A. (2015). The PREP pipeline: Standardized preprocessing for large-scale EEG analysis. Front. Neuroinf..

[17] Mullen T.R., Kothe C.A.E., Chi Y.M., Ojeda A., Kerth T., Makeig S., Jung T.-P., Cauwenberghs G. (2015). Real-Time Neuroimaging and Cognitive Monitoring Using Wearable Dry EEG. IEEE Trans. Biomed. Eng..

[18] Blum S., Jacobsen N.S.J., Bleichner M.G., Debener S. (2019). A riemannian modification of artifact subspace reconstruction for EEG artifact handling. Front. Hum. Neurosci..

[19] Piazza C., Miyakoshi M., Akalin-Acar Z., Cantiani C., Reni G., Bianchi A.M., Makeig S., Kyriacou E., Christofides S., Pattichis C.S. (2016). An Automated Function for Identifying EEG Independent Components Representing Bilateral Source Activity. Proceedings of the XIV Mediterranean Conference on Medical and Biological Engineering and Computing.

[20] Pion-Tonachini L., Kreutz-Delgado K., Makeig S. (2019). ICLabel: An automated electroencephalographic independent component classifier, dataset, and website. Neuroimage.

[21] Rousseeuw P.J. (1987). Silhouettes: A graphical aid to the interpretation and validation of cluster analysis. J. Comput. Appl. Math..

[22] Groppe D.M., Urbach T.P., Kutas M. (2011). Mass univariate analysis of event-related brain potentials/fields I: A critical tutorial review. Psychophysiology.

[23] Esfahani E.T., Sundararajan V. (2011). Using brain–computer interfaces to detect human satisfaction in human–robot interaction. Int. J. Human. Robot..

[24] Park W., Ki D., Kim D.H., Kwon G.H., Kim S.P., Kim L. EEG correlates of user satisfaction of haptic sensation. Proceedings of the 2015 IEEE International Conference on Consumer Electronics (ICCE).

[25] Park W., Kim D.-H., Kim S.-P., Lee J.-H., Kim L. (2018). Gamma EEG correlates of haptic preferences for a dial interface. IEEE Access.

[26] Song M., Kim J. (2019). A Paradigm to Enhance Motor Imagery Using Rubber Hand Illusion Induced by Visuo-Tactile Stimulus. IEEE Trans. Neural Syst. Rehabil. Eng..

[27] Haggard P. (2017). Sense of agency in the human brain. Nat. Rev. Neurosci..

[28] Wen W. (2019). Does delay in feedback diminish sense of agency? A review. Conscious Cogn..

[29] Kawabe T. (2013). Inferring sense of agency from the quantitative aspect of action outcome. Conscious Cogn..

[30] Kim H., Kim Y., Miyakoshi M., Stapornchaisit S., Yoshimura N., Koike Y. (2021). Brain Activity Reflects Subjective Response to Delayed Input When Using an Electromyography-Controlled Robot. Front. Syst. Neurosci..

[31] Engel A.K., Singer W. (2001). Temporal binding and the neural correlates of sensory awareness. Trends Cogn. Sci. (Regul. Ed.).

[32] Rieder M.K., Rahm B., Williams J.D., Kaiser J. (2011). Human γ-band activity and behavior. Int. J. Psychophysiol..

[33] Matsumoto A., Ichikawa Y., Kanayama N., Ohira H., Iidaka T. (2006). Gamma band activity and its synchronization reflect the dysfunctional emotional processing in alexithymic persons. Psychophysiology.

